# 4-Bromo-2-{[(pyridin-3-ylmeth­yl)imino]­meth­yl}phenol

**DOI:** 10.1107/S1600536811003011

**Published:** 2011-01-29

**Authors:** Kwang Ha

**Affiliations:** a School of Applied Chemical Engineering, The Research Institute of Catalysis, Chonnam National University, Gwangju 500-757, Republic of Korea

## Abstract

The title compound, C_13_H_11_BrN_2_O, is a polydentate Schiff base and reveals intra­molecular O—H⋯N hydrogen bonding between the hy­droxy O atom and the imino N atom. The dihedral angle between the aromatic ring and the pyridyl ring is 71.7 (1)°. In the crystal, the mol­ecules are stacked in columns along the *c* axis and several inter­molecular π–π inter­actions are present between the six-membered rings, with a shortest centroid–centroid distance of 3.707 (2) Å.

## Related literature

For the crystal structure of 4-bromo-2-{[(pyridin-2-ylmethyl)imino]methyl}phenol, see: Zhang *et al.* (2003 ▶). 
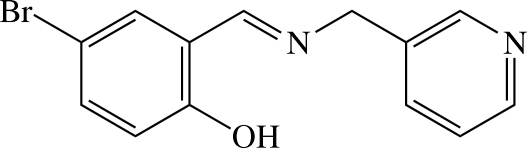


## Experimental

### 

#### Crystal data

C_13_H_11_BrN_2_O
*M* *_r_* = 291.15
Monoclinic, 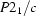

*a* = 14.0947 (19) Å
*b* = 6.0994 (8) Å
*c* = 14.0373 (19) Å
β = 103.704 (3)°
*V* = 1172.4 (3) Å^3^
*Z* = 4
Mo *K*α radiation
μ = 3.49 mm^−1^
*T* = 200 K
0.30 × 0.22 × 0.14 mm

#### Data collection

Bruker SMART 1000 CCD diffractometer
Absorption correction: multi-scan (*SADABS*; Bruker, 2000 ▶) *T*_min_ = 0.833, *T*_max_ = 1.000
8297 measured reflections
2906 independent reflections
1859 reflections with *I* > 2σ(*I*)
*R*_int_ = 0.037

#### Refinement

*R*[*F* ^2^ > 2σ(*F* ^2^)] = 0.037
*wR*(*F* ^2^) = 0.099
*S* = 1.13
2906 reflections
158 parameters
H atoms treated by a mixture of independent and constrained refinement
Δρ_max_ = 0.53 e Å^−3^
Δρ_min_ = −0.84 e Å^−3^

### 

Data collection: *SMART* (Bruker, 2000 ▶); cell refinement: *SAINT* (Bruker, 2000 ▶); data reduction: *SAINT*; program(s) used to solve structure: *SHELXS97* (Sheldrick, 2008 ▶); program(s) used to refine structure: *SHELXL97* (Sheldrick, 2008 ▶); molecular graphics: *ORTEP-3* (Farrugia, 1997 ▶) and *PLATON* (Spek, 2009 ▶); software used to prepare material for publication: *SHELXL97*.

## Supplementary Material

Crystal structure: contains datablocks global, I. DOI: 10.1107/S1600536811003011/ng5109sup1.cif

Structure factors: contains datablocks I. DOI: 10.1107/S1600536811003011/ng5109Isup2.hkl

Additional supplementary materials:  crystallographic information; 3D view; checkCIF report

## Figures and Tables

**Table 1:** Hydrogen-bond geometry (Å, °)

*D*—H⋯*A*	*D*—H	H⋯*A*	*D*⋯*A*	*D*—H⋯*A*
O1—H1⋯N1	0.96 (5)	1.80 (5)	2.616 (5)	141 (5)

**Fig. 1.**
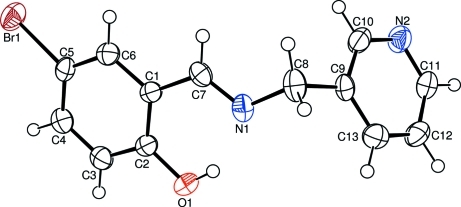
The structure of the title compound, with displacement ellipsoids drawn at the 50% probability level.

**Fig. 2.**
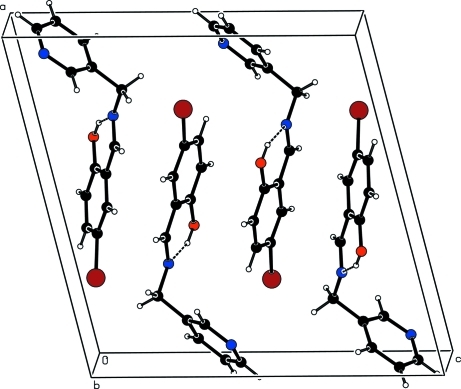
View of the unit-cell contents of the title compound. Hydrogen-bond interactions are drawn with dashed lines.

## supplementary crystallographic information

### Comment

The title compound, C_13_H_11_BrN_2_O, is a polydentate Schiff base (Fig. 1), which can act as a monobasic bi- or tridentate ligand, that is, the NO or N_2_O donor atoms can coordinate to a metal ion or metal ions. The compound crystallized in the monoclinic space group *P*2_1_/*c*, whereas the previously reported analogous compound 4-bromo-2-{[(pyridin-2-ylmethyl)imino]methyl}phenol crystallized in the triclinic space group *P*1 (Zhang *et al.*, 2003).

The Schiff base reveals intramolecular O—H···N hydrogen bonding between the hydroxy O atom and the imino N atom with d(O···N) = 2.616 (5) Å forming a nearly planar six-membered ring (Fig. 2, Table 1). The O1—H1 bond length is somewhat long, but the zwitterionic possibility can be excludued. The dihedral angle between the benzene ring and the pyridine ring is 71.7 (1)°. The N1—C7/8 bond lengths and the C7—N1—C8 bond angle indicate that the imino N1 atom is *sp*^2^-hybridized [*d*(N1═C7) = 1.271 (5) Å and d(N1—C8) = 1.468 (5) Å; <C7—N1—C8 = 117.6 (4)°]. In the crystal structure, the molecules are stacked in columns along the *c* axis and several intermolecular π-π interactions are present between the six-membered rings, with a shortest centroid-centroid distance of 3.707 (2) Å.

### Experimental

3-(Aminomethyl)pyridine (1.0751 g, 9.942 mmol) and 5-bromosalicylaldehyde (1.9988 g, 9.943 mmol) in EtOH (20 ml) were stirred for 2 h at room temperature. After add of pentane (50 ml) to the solution, the formed precipitate at -85 °C was then separated by filtration, washed with pentane, and dried at 50 °C, to give a gold-yellow powder (2.7240 g). Crystals suitable were obtained by slow evaporation from a CH_3_CN solution.

### Refinement

H atoms were positioned geometrically and allowed to ride on their respective parent atoms [C—H = 0.95 Å (CH) or 0.99 Å (CH_2_) and *U*_iso_(H) = 1.2*U*_eq_(C)]. The hydroxy H atom was located from Fourier difference maps and refined isotropically [O—H = 0.96 (5) Å].

### Crystal data

**Table:**

C_13_H_11_BrN_2_O	*F*(000) = 584
*M**_r_* = 291.15	*D*_x_ = 1.649 Mg m^−^^3^
Monoclinic, *P*2_1_/*c*	Mo *K*α radiation, λ = 0.71073 Å
Hall symbol: -P 2ybc	Cell parameters from 3009 reflections
*a* = 14.0947 (19) Å	θ = 3.0–27.0°
*b* = 6.0994 (8) Å	µ = 3.49 mm^−^^1^
*c* = 14.0373 (19) Å	*T* = 200 K
β = 103.704 (3)°	Block, yellow
*V* = 1172.4 (3) Å^3^	0.30 × 0.22 × 0.14 mm
*Z* = 4	

### Data collection

**Table:**

Bruker SMART 1000 CCD diffractometer	2906 independent reflections
Radiation source: fine-focus sealed tube	1859 reflections with *I* > 2σ(*I*)
graphite	*R*_int_ = 0.037
φ and ω scans	θ_max_ = 28.3°, θ_min_ = 3.0°
Absorption correction: multi-scan (*SADABS*; Bruker, 2000)	*h* = −18→17
*T*_min_ = 0.833, *T*_max_ = 1.000	*k* = −8→8
8297 measured reflections	*l* = −13→18

### Refinement

**Table:**

Refinement on *F*^2^	Primary atom site location: structure-invariant direct methods
Least-squares matrix: full	Secondary atom site location: difference Fourier map
*R*[*F*^2^ > 2σ(*F*^2^)] = 0.037	Hydrogen site location: inferred from neighbouring sites
*wR*(*F*^2^) = 0.099	H atoms treated by a mixture of independent and constrained refinement
*S* = 1.13	*w* = 1/[σ^2^(*F*_o_^2^) + (0.0134*P*)^2^ + 2.5694*P*] where *P* = (*F*_o_^2^ + 2*F*_c_^2^)/3
2906 reflections	(Δ/σ)_max_ < 0.001
158 parameters	Δρ_max_ = 0.53 e Å^−^^3^
0 restraints	Δρ_min_ = −0.84 e Å^−^^3^

### Special details

**Table:**

Geometry. All e.s.d.'s (except the e.s.d. in the dihedral angle between two l.s. planes) are estimated using the full covariance matrix. The cell e.s.d.'s are taken into account individually in the estimation of e.s.d.'s in distances, angles and torsion angles; correlations between e.s.d.'s in cell parameters are only used when they are defined by crystal symmetry. An approximate (isotropic) treatment of cell e.s.d.'s is used for estimating e.s.d.'s involving l.s. planes.
Refinement. Refinement of *F*^2^ against ALL reflections. The weighted *R*-factor *wR* and goodness of fit *S* are based on *F*^2^, conventional *R*-factors *R* are based on *F*, with *F* set to zero for negative *F*^2^. The threshold expression of *F*^2^ > σ(*F*^2^) is used only for calculating *R*-factors(gt) *etc*. and is not relevant to the choice of reflections for refinement. *R*-factors based on *F*^2^ are statistically about twice as large as those based on *F*, and *R*- factors based on ALL data will be even larger.

### Fractional atomic coordinates and isotropic or equivalent isotropic displacement parameters (Å^2^)

**Table:**

	*x*	*y*	*z*	*U*_iso_*/*U*_eq_	
Br1	0.76468 (3)	0.70338 (8)	0.44621 (4)	0.04693 (16)	
O1	0.3873 (2)	0.1972 (5)	0.3696 (2)	0.0395 (7)	
H1	0.332 (4)	0.283 (9)	0.338 (4)	0.081 (18)*	
N1	0.3004 (2)	0.5487 (6)	0.2840 (2)	0.0353 (8)	
N2	0.0887 (2)	0.9342 (6)	0.4172 (3)	0.0416 (9)	
C1	0.4726 (3)	0.5284 (6)	0.3484 (3)	0.0267 (8)	
C2	0.4714 (3)	0.3123 (6)	0.3834 (3)	0.0281 (8)	
C3	0.5585 (3)	0.2149 (7)	0.4341 (3)	0.0338 (9)	
H3	0.5578	0.0687	0.4574	0.041*	
C4	0.6450 (3)	0.3279 (7)	0.4507 (3)	0.0338 (9)	
H4	0.7038	0.2606	0.4855	0.041*	
C5	0.6462 (3)	0.5405 (7)	0.4164 (3)	0.0307 (9)	
C6	0.5623 (3)	0.6397 (7)	0.3654 (3)	0.0307 (9)	
H6	0.5649	0.7845	0.3413	0.037*	
C7	0.3835 (3)	0.6408 (7)	0.2984 (3)	0.0307 (9)	
H7	0.3876	0.7867	0.2761	0.037*	
C8	0.2146 (3)	0.6772 (8)	0.2349 (3)	0.0412 (10)	
H8A	0.1805	0.6019	0.1740	0.049*	
H8B	0.2358	0.8231	0.2169	0.049*	
C9	0.1454 (2)	0.7050 (7)	0.3019 (3)	0.0317 (8)	
C10	0.1465 (3)	0.8949 (7)	0.3555 (3)	0.0391 (10)	
H10	0.1915	1.0061	0.3483	0.047*	
C11	0.0261 (3)	0.7758 (7)	0.4251 (3)	0.0378 (10)	
H11	−0.0164	0.7995	0.4675	0.045*	
C12	0.0194 (3)	0.5796 (7)	0.3754 (3)	0.0385 (10)	
H12	−0.0261	0.4709	0.3842	0.046*	
C13	0.0800 (3)	0.5436 (7)	0.3127 (3)	0.0373 (10)	
H13	0.0766	0.4098	0.2774	0.045*	

### Atomic displacement parameters (Å^2^)

**Table:**

	*U*^11^	*U*^22^	*U*^33^	*U*^12^	*U*^13^	*U*^23^
Br1	0.0307 (2)	0.0546 (3)	0.0530 (3)	−0.0087 (2)	0.00510 (18)	0.0004 (2)
O1	0.0347 (15)	0.0333 (16)	0.0524 (19)	−0.0090 (13)	0.0140 (14)	0.0004 (15)
N1	0.0286 (16)	0.043 (2)	0.0354 (19)	0.0039 (15)	0.0092 (15)	−0.0030 (16)
N2	0.0347 (18)	0.046 (2)	0.047 (2)	−0.0025 (16)	0.0159 (17)	−0.0081 (18)
C1	0.0302 (19)	0.027 (2)	0.0246 (19)	−0.0023 (15)	0.0093 (16)	−0.0021 (15)
C2	0.0327 (19)	0.030 (2)	0.0245 (19)	−0.0043 (17)	0.0121 (16)	−0.0047 (17)
C3	0.041 (2)	0.033 (2)	0.032 (2)	0.0032 (18)	0.0174 (18)	0.0026 (18)
C4	0.035 (2)	0.040 (3)	0.028 (2)	0.0048 (18)	0.0087 (17)	0.0010 (18)
C5	0.0261 (18)	0.038 (2)	0.030 (2)	−0.0020 (16)	0.0106 (16)	−0.0028 (17)
C6	0.035 (2)	0.031 (2)	0.028 (2)	−0.0013 (16)	0.0114 (17)	0.0003 (16)
C7	0.035 (2)	0.033 (2)	0.026 (2)	0.0058 (17)	0.0119 (17)	−0.0034 (16)
C8	0.031 (2)	0.057 (3)	0.035 (2)	0.010 (2)	0.0053 (18)	0.002 (2)
C9	0.0216 (17)	0.040 (2)	0.031 (2)	0.0056 (17)	0.0015 (15)	−0.0001 (19)
C10	0.029 (2)	0.042 (3)	0.046 (3)	−0.0068 (18)	0.0092 (19)	−0.004 (2)
C11	0.0271 (19)	0.050 (3)	0.038 (2)	−0.0008 (19)	0.0098 (17)	−0.001 (2)
C12	0.034 (2)	0.042 (3)	0.041 (2)	−0.0079 (19)	0.0106 (19)	0.006 (2)
C13	0.039 (2)	0.036 (2)	0.033 (2)	−0.0008 (19)	0.0008 (18)	−0.0017 (18)

### Geometric parameters (Å, °)

**Table:**

Br1—C5	1.903 (4)	C5—C6	1.369 (5)
O1—C2	1.352 (4)	C6—H6	0.9500
O1—H1	0.96 (5)	C7—H7	0.9500
N1—C7	1.271 (5)	C8—C9	1.517 (5)
N1—C8	1.468 (5)	C8—H8A	0.9900
N2—C11	1.331 (5)	C8—H8B	0.9900
N2—C10	1.344 (5)	C9—C10	1.379 (6)
C1—C6	1.406 (5)	C9—C13	1.381 (5)
C1—C2	1.408 (5)	C10—H10	0.9500
C1—C7	1.456 (5)	C11—C12	1.377 (6)
C2—C3	1.397 (5)	C11—H11	0.9500
C3—C4	1.372 (5)	C12—C13	1.382 (6)
C3—H3	0.9500	C12—H12	0.9500
C4—C5	1.384 (5)	C13—H13	0.9500
C4—H4	0.9500		
			
C2—O1—H1	112 (3)	C1—C7—H7	119.2
C7—N1—C8	117.6 (4)	N1—C8—C9	110.4 (3)
C11—N2—C10	116.1 (4)	N1—C8—H8A	109.6
C6—C1—C2	118.5 (3)	C9—C8—H8A	109.6
C6—C1—C7	119.5 (3)	N1—C8—H8B	109.6
C2—C1—C7	121.9 (3)	C9—C8—H8B	109.6
O1—C2—C3	119.1 (4)	H8A—C8—H8B	108.1
O1—C2—C1	121.2 (3)	C10—C9—C13	117.4 (4)
C3—C2—C1	119.6 (3)	C10—C9—C8	120.4 (4)
C4—C3—C2	120.7 (4)	C13—C9—C8	122.2 (4)
C4—C3—H3	119.6	N2—C10—C9	124.8 (4)
C2—C3—H3	119.6	N2—C10—H10	117.6
C3—C4—C5	119.7 (4)	C9—C10—H10	117.6
C3—C4—H4	120.2	N2—C11—C12	123.8 (4)
C5—C4—H4	120.2	N2—C11—H11	118.1
C6—C5—C4	121.0 (4)	C12—C11—H11	118.1
C6—C5—Br1	119.2 (3)	C11—C12—C13	118.8 (4)
C4—C5—Br1	119.7 (3)	C11—C12—H12	120.6
C5—C6—C1	120.4 (4)	C13—C12—H12	120.6
C5—C6—H6	119.8	C9—C13—C12	119.1 (4)
C1—C6—H6	119.8	C9—C13—H13	120.5
N1—C7—C1	121.7 (4)	C12—C13—H13	120.5
N1—C7—H7	119.2		
			
C6—C1—C2—O1	179.8 (3)	C6—C1—C7—N1	−178.0 (4)
C7—C1—C2—O1	1.8 (5)	C2—C1—C7—N1	0.0 (5)
C6—C1—C2—C3	0.4 (5)	C7—N1—C8—C9	−118.3 (4)
C7—C1—C2—C3	−177.6 (3)	N1—C8—C9—C10	98.1 (5)
O1—C2—C3—C4	−179.0 (3)	N1—C8—C9—C13	−81.3 (5)
C1—C2—C3—C4	0.4 (5)	C11—N2—C10—C9	−0.4 (6)
C2—C3—C4—C5	−0.3 (6)	C13—C9—C10—N2	−0.1 (6)
C3—C4—C5—C6	−0.6 (6)	C8—C9—C10—N2	−179.5 (4)
C3—C4—C5—Br1	176.2 (3)	C10—N2—C11—C12	0.8 (6)
C4—C5—C6—C1	1.3 (6)	N2—C11—C12—C13	−0.7 (6)
Br1—C5—C6—C1	−175.5 (3)	C10—C9—C13—C12	0.2 (6)
C2—C1—C6—C5	−1.2 (5)	C8—C9—C13—C12	179.6 (4)
C7—C1—C6—C5	176.8 (3)	C11—C12—C13—C9	0.2 (6)
C8—N1—C7—C1	178.6 (3)		

### Hydrogen-bond geometry (Å, °)

**Table:**

*D*—H···*A*	*D*—H	H···*A*	*D*···*A*	*D*—H···*A*
O1—H1···N1	0.96 (5)	1.80 (5)	2.616 (5)	141 (5)
